# Grape breeding is a key link in the development of the grapes and wine-making industry

**DOI:** 10.18699/VJ21.045

**Published:** 2021-07

**Authors:** E.A. Egorov

**Affiliations:** North Caucasian Federal Scientific Center of Horticulture, Viticulture, Wine-Making, Krasnodar, Russia

**Keywords:** grapes, breeding, genetic resources, methods, varieties, breeding achievements, seedlings, introduction, scientific and practical problems, виноград, селекция, генетические ресурсы, методы, сорта, селекционные достижения, cаженцы, интродукция, научно-практические проблемы.

## Abstract

The article considers the legislative and regulatory acts that specify the tasks in the implementation of breeding processes. The results of the creation, variety testing, patenting and introduction of grape varieties and clones into the State Register of the Russian Federation for 2010–2020 are presented. The article analyzes the relationship between the indicators of industrial development with the production volumes of planting material, the use of domestic varieties that are included in the State Register of the Russian Federation. The characteristic of ampelographic collections – the genetic resources of grapes – is given. A comparative analysis of many years’ worth of data on the assessment of the adaptive potential of domestic varieties and introduced varieties is presented. The article describes domestic varieties, from which premium wines are produced, which not only competes with European varieties, but also surpasses the organoleptic properties and biochemical parameters of grape must and wine material. The main problems hindering the wide demand for domestic varieties on the market, including a substantial amount of imported European varietal planting material, are described. The necessity of accelerating breeding processes is actualized, modern methods are identified, including those of generative and genomic selection, transgenic technologies, cellular, mutational, and clone selection, and priority areas in breeding are presented. The numerical and qualitative analyses of the composition of breeding scientists is given, the trends of increasing the number and qualitative composition of breeders, the influx of young people, the growing need for training qualified personnel are noted. The number of bachelor’s, master’s and post-graduate students specializing in viticulture in general and in selection in particular as well as the number of defended dissertation studies on grape breeding has been found to be insufficient. The main scientific and practical problems in the organization and implementation of breeding processes in ensuring the development of the industry are updated, including a low share of domestic varieties in the produced planting material and planting, the lack of a systemically implemented varietal and technological policy, the imperfection of the legal system for the protection of intellectual property, a low availability of instrumentation and analytical equipment for the implementation of breeding by modern methods.

## Abstract (ru)

Рассмотрены законодательные и нормативные акты, конкретизирующие задачи в осуществлении селекционных процессов. Представлены результаты создания, сортоиспытания, патентования и введения в Государственный реестр Российской Федерации сортов и клонов винограда за период 2010–2020 гг. Проанализирована взаимосвязь показателей отраслевого развития с объемами производства посадочного материала, использованием сортов отечественной селекции, составляющих предмет Государственного реестра РФ. Приведены характеристика ампелографических коллекций – генетических ресурсов винограда, и сопоставимый анализ многолетних данных по оценке адаптивного потенциала сортов отечественной селекции и сортов-интродуцентов. Охарактеризованы сорта отечественной селекции, из которых производятся вина класса премиум; они не только составляют конкуренцию сортам европейской селекции, но и превосходят их по органолептическим свойствам и биохимическим показателям виноградного сусла и виноматериала. Обозначены основные проблемы, сдерживающие широкую востребованность рынком сортов винограда отечественной селекции, к числу которых следует отнести значительный объем импорта европейского сортового посадочного материала. Подчеркнута необходимость ускорения селекционных процессов, обозначены современные методы, в том числе генеративная и геномная селекция, трансгенные технологии, клеточная, мутационная, клоновая селекция. Представлены приоритетные направления в селекции. Приводятся и анализируются сведения о количественном и качественном составе ученых-селекционеров, отмечены тенденции увеличения их численности и качественного состава, притока молодежи, растущей потребности в подготовке квалифицированных кадров. Актуализированы основные научно-практические проблемы в организации и осуществлении селекционных процессов в обеспечение развития отрасли, среди которых низкая доля сортов отечественной селекции в производимом посадочном материале и закладке насаждений, отсутствие системно реализуемой сортовой и технологической политики, несовершенство правовой системы по охране интеллектуальной собственности, низкая обеспеченность приборно-аналитическим оснащением для осуществления селекционных процессов современными методами.

## Introduction

The development of the grapes and wine-making industry is one of the priorities in the modern agricultural policy of the Russian Federation and is intended to increase not only the volume of production of high-quality products and equivalent import substitution, but also its own resource and technological support. One of the Subprograms prepared for approval of the Federal Scientific and Technical Program for Development of Agriculture for 2017–2025, approved by the Decree of the Government of the Russian Federation No. 996 of August 25, 2017, is the “Development of Viticulture, Including Nursery Farming”.

In its goal setting, the Subprogram focuses on ensuring the growth of viticulture production volumes based on improving the assortment of grapes (primarily varieties and clones of domestic breeding), using domestic virus-free certified planting material. Complex research programs and complex scientific and technical projects, which are elements of the Subprogram, specify the volume of breeding work carried out by scientific and educational institutions, based on the needs of the industry for its further development (Egorov et al., 2020). 

Federal Law No. 468-FZ “On Viticulture and Winemaking in the Russian Federation” was passed in December 2019 and was enacted in July 2020. The Law is a legislative act harmonized with similar European laws, the basis for legal, organizational, technological and economic regulation in the field of efficient production, turnover and consumption of grape growing and wine-making products. The Federal Law also directs the grapes and wine-making branch to determine the issues of import substitution, including the breeding of domestic varieties and clones, the production of planting material of the highest quality categories, and the laying of plantings mainly with planting material of domestic production. 

The passed legislation and regulations, which specify the most urgent tasks of industry development, orient the breeding processes to the accelerated creation of varieties, the selection of clones of varieties (variety improvement) on the basis of modern methods in order to bring their quality characteristics into compliance with the requirements of production, technical regulations, international and national standards. 

## Results and discussion

The comprehensive research program of the Subprogram provides for the creation of 12 grape varieties and clones for the period 2020–2025: North Caucasian Federal Scientific Center of Horticulture, Viticulture, Wine-Making (NCFSCHVW)* – 6, All-Russian National Scientific Research Institute of Vine and Winemaking “Magarach” RAS (ARNSRIVW “Magarach”) – 3, All-Russian Scientific Research Ya.I. Potapenko Institute for Viticulture and Winemaking – Branch of the Federal Rostov Agricultural Research Centre (ARSRIVW – Branch of FRARC) – 3. A complex scientific and technical project provides for the creation of 6 varieties and clones of grapes during the same period, according to the available applications of economic entities. 

* The structural composition of the NCFSCHVW includes the branches of the Anapa Zonal Experimental Station of Viticulture and Winemaking (AZESVW) and the Dagestan Selection Testing Station of Viticulture and Horticulture (DSTSVH).


Until the present, the work on the breeding of grapes in the region has been carried out by scientific and educational organizations within the framework of the North Caucasian Center’s Program for the Breeding of Fruit, Berry, Nut-fruited, Ornamental Crops and Grapes for the period up to 2030, passed by the coordination meeting on August 27, 2013. In order to ensure coordinated actions in the implementation of the Program, a Scientific Coordinating Council, which is the coordinating body, was established. It is formed from the leading scientists-breeders of scientific and educational institutions that are part of the area of activity of the North Caucasian Center for Breeding. 

The corporate Program passed in 2013 provided for the creation of 35 grape varieties for the period up to 2030. Scientific institutions in the South of Russia created and submitted to the State Variety Testing a significant number of varieties that were entered into the State Register of the Russian Federation during 2010–2020 (see the Table).

**Table 1.**
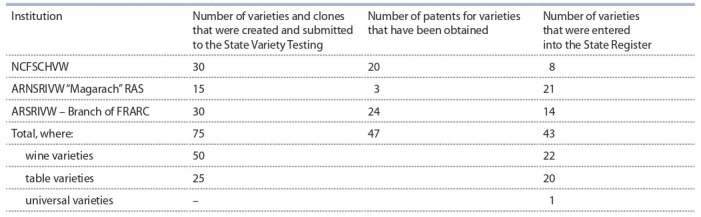
Creation of grape varieties and clones by scientific institutions of the South of Russia for 2010–2020

The performed breeding processes for the creation of new varieties and variety improvement (selection of clones) should ensure the progressive development of the industry as a whole and the production of young plants from improved source plants with varietal identification at the gene level.

Due to natural and climatic features, grape growing is concentrated in the subjects of the Southern and North Caucasus Federal District (97.5 %): 27.5 thousand hectares or 28.7 % of the area of grape plantations of the Russian Federation are in the Krasnodar Region; 25.9 thousand hectares or 27.0 % – in the Republic of Dagestan; 25.7 thousand hectares or 26.8 % – in the Republic of Crimea and Sevastopol; 5.9 thousand hectares or 6.2 % – in the Stavropol Territory 4 thousand hectares or 4.1 % – in the Rostov Region (Egorov et al., 2018b).

The positive dynamics in the development of the grapes and wine branch over the past ten years should be noted: the total area of grape plantations in the Russian Federation increased by 33.8 thousand hectares, or an average of 5 % per year; the increase in whole yields amounted to 342.2 thousand tons, or 8.1 % per year; the yield increased by 22.2 centner/ha, or 1.3 times, due to the application of modern agricultural technologies, grape varieties most adapted to the edaphoclimatic conditions of cultivation (Egorov et al., 2020). 

4.3 thousand hectares of grape plantations are laid annually in the Russian Federation, the largest area of laying in the Krasnodar Region is 1.5 thousand hectares (34.9 % of the level of the Russian Federation), the Republic of Dagestan – 1.4 thousand hectares (32.6 % of the level of the Russian Federation), the Republic of Crimea, including the city of Sevastopol – 0.84 thousand hectares (19.5 %).

The total annual requirement of planting material for the implementation of planned laying (on average more than 5.0 thousand hectares per year), repairs (on average 2 %) and renovation of grape plantations (with a renovation rate of 5 %) is more than 17.8 million pcs or 250 % of the actual production; the requirement of planting material will be more than 80 million pcs by 2025. The laying of plantings is provided by young plants of domestic production only by 50 %

An important trend of ensuring the food security of the Russian Federation is to reduce dependence on imported grape planting material through the production of domestic young plants and the creation of varieties of domestic breeding with integrated technological equipment for the breeding process. 

The basis of the breeding process is plant genetic resources. In Russia, the main holders of grape collections are AZESVW – Branch of NCFSCHVW – 5001, ARNSRIVW “Magarach” – 4620 samples, ARSRIVW – Branch of FRARC– 895 samples, DSTSVH – Branch of NCFSCHVW– 800 samples. The total number of cultivars in the collections is 10 526 pieces, and it has a positive trend: over the past 10 years, it has increased by 18 %, from 8860 to 10 526 pcs. The expansion of the collections to 11 316 samples is expected by 2025 (Egorov et al., 2018a).

Varieties and hybrid forms of Russian ampelographic collections are collected from more than 40 countries. The largest number of varieties are from Russia, as well as from Moldova, Uzbekistan, France, Georgia, Greece, Ukraine, Hungary, the United States, Armenia, the Czech Republic, Japan and other countries

The samples of the collection are studied both by traditional methods to specificate the sources of breeding valuable traits, and by molecular genetic methods to identify donors of valuable genes for use in the breeding of new most popular varieties (Ilnitskaya, Makarkina, 2016). 

Much attention has been paid to the study of autochthonous varieties in recent years, according to the results of the conducted studies; the following varieties have been identified as promising for high-quality winemaking: Makhrovatchik, Belobulanyi, Tsimladar, Sypun Chernyi. Research in this direction continues (Egorov, Petrov, 2020).

**Fig. 1.**
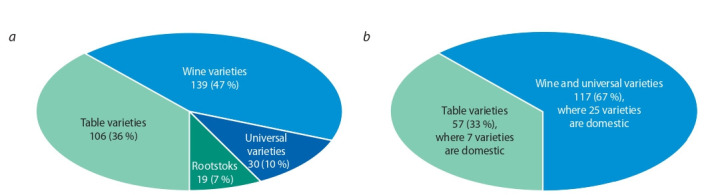
Structure of grape varieties and clones approved (a) and used (b) in industrial plantings in Russia.

According to the “State Register of Selection Achievements…” (2020) 294 varieties are allowed to be used in industrial plantings in Russia in 2020. There are 180 units of domestic varieties and clones accounts, which is 65.5 % (Fig. 1, a). The industrial plantings are dominated mainly by Western European varieties (see Fig. 1, b). Domestic and autochthonous varieties account for less than 1 % of each genotype. As a result of the dominance of introduced varieties in grape plantations, there is a decrease in the level of realization of the potential of economic productivity of grapes (up to 60 % in the Krasnodar Region), as well as the agrobiological and environmental stability of grape agrocenoses under the influence of biotic and abiotic stressors (Petrov, 2016). 

Since all the biological and economically valuable characteristics of a given variety are better realized in their places of origin, autochthonous and domestic varieties, in contrast to the introduced varieties, are characterized by high adaptability, productivity and quality (Ilnitskaya et al., 2018). 

A comparative analysis of long-term data on the assessment of the adaptive potential of grapes indicates that it is significantly higher in domestic varieties than in introduced varieties: for example, the amount of evolved buds after wintering in introduced varieties averaged 87 %, and the yield is 110.9 centner/ha, while in domestic varieties the amount of evolved buds averaged 94 %, the yield is 128.9 centner/ha.

**Fig. 2.**
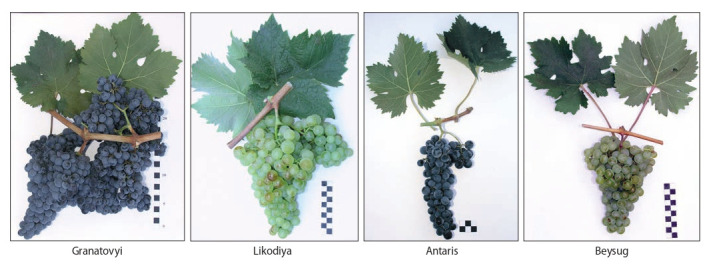
Varieties of the NCFSCHVW breeding

A number of varieties of domestic breeding are worthy of competition with classic European varieties (Fig. 2): in particular, the Granatovyi variety (NCFSCHVW breeding) is competitive with the Cabernet-Sauvignon variety for the production of high-quality table and liqueur wines. Having high indicators of crop quality, Granatovyi variety surpasses Cabernet-Sauvignon in yield (130 centner/ha versus 90 centner/ha), parameters of resistance to fungal diseases, organoleptic properties and biochemical parameters of grape must and wine materials (Petrov et al., 2012)

The lack of modern infrastructure and outdated material and technical base of organizations participating in breeding and nursery production, the aggressive position of distributors of planting material of foreign varieties, the lack of a systemically implemented varietal and technological policy, combined with insufficient measures of state support, are the main reasons that most domestic varieties are not in demand. Specifically, the import of European varietal planting material increased from 4.4 million pcs to 15.4 million pcs for the period 2010–2020, the provision of bookmarks with imported planting material was 38 % in 2010, 56 % in 2019.

The Civil Code of the Russian Federation (Part four) of 18.12.2006 No. 230-FZ (eds. on 31.07.2020) establishes the right to a breeding achievement, by which the object of the right is recognized and protected subject to state registration of the breeding achievement in the State Register of Protected Breeding Achievements, in accordance with which the federal executive authority for breeding achievements issues the applicant a patent for a breeding achievement. 

The exclusivity period and the patent certifying this right is thirty-five years for grape varieties. The specific responsibility of the patent owner of a breeding achievement as an object of intellectual property is their duty to maintain the variety during the term of the patent, in a way that the characteristics specified in the description of the variety, breed, compiled at the time of registration in the register are preserved. 

The need for the permission of the patent owner to use the breeding achievement (conclusion of a license agreement) is established, in particular, in the production and reproduction of the variety. 

Article 1446 of the Civil Code of the Russian Federation prescribes actions that violate the rights of the author of a breeding achievement or other patent owner; however, measures of influence against agricultural producers who violate the rights of the patent owner are not regulated. The federal executive authority in the field of registration of rights on a breeding achievement is the Federal State Budgetary Institution “Gossortcommission”.

The grape was included in the “List of genera and species for which the economic utility of the variety is evaluated according to the results of state tests” until 2019. The state tests in the North Caucasus region of admission (6) were conducted at eight state strain-trial stations (Blagodarnenskiy, Volgodonskiy, Levokumskiy, Prokhladnenskiy, Rostovskiy, Sudakskiy, Khasavyurtovskiy and Anapskiy), evaluating new grape varieties; in the Lower Volga region of admission – at one state strain-trial station (Astarakhanskiy). Variety tests were carried without charge.

The grape was transferred to list B “The list of genera and species for which the economic utility of the variety is evaluated by expert assessment” in 2019, according to which it is necessary to place a mandatory laying of the variety to confirm the distinguishability, uniformity and stability on the Sudak state strain-trial station (at least 7 young plants) and the presence of plantings of the grape variety with an area of at least one hectare with the provision of yield data for at least three years. Expert evaluation is carried out on a fee basis.

The main reasons for the long duration of the breeding process of grapes are a certain complexity in conducting genetic research and carrying out breeding work, the imperfection of the scientific and technical base for conducting research. The duration of the process of creating a variety with varietal tests before its inclusion in the State Register of the Russian Federation is 25 years in accordance with the existing regulations, which, in turn, actualizes the need to accelerate the breeding process. 

Currently, the creation of new grape genotypes can be carried out by generative breeding, transgenic technologies, cell and mutation breeding, as well as clone selection in grape plantations (Petrov, Ilnitskaya, 2017). The combined use of generative breeding and DNA marker selection or genomic breeding shows high efficiency. The use of DNA markers in breeding work is most effective in virtue of necessity to combine a number of genes (for example, genes that control plant resistance to pathogens) or due to the manifestation of a trait that can be evaluated only when the bushes enter fruiting (for example, the trait of seedless berries). The active use of this approach in the practice of the world’s leading centers of grape breeding indicates the prospects of this direction for domestic science.

Additionally, a significant direction in the grapes breeding is clonal selection, which can be considered as a way of variety improvement, the allocation of more adapted genotypes to specific agro-climatic conditions with a higher level of productivity and quality of grapes. Clone selection is very important for introduced varieties, given that their long-term use in domestic agroecological conditions leads to mutations that reduce the economically valuable and biological characteristics of the varieties. 

Currently, breeders pay special attention to the combination of traits of complex resistance to biotic and abiotic environmental factors in one genotype in combination with stable yield and high quality of grapes (Ilnitskaya et al., 2016).

The main priority characteristics of marketability for table grape varieties are large berry, elegant cluster, good taste, seedlessness, transportability and storability. The early ripening period is also a valuable feature – as usual, the price for the harvest of early varieties reaches the maximum values.

The quality requirements for wine grape varieties are based on the characteristics of the types and brands of wines for which they can be used. In general, the main task in the breeding of wine varieties is to preserve the quality of classic European varieties and at the same time increase resistance to dominant pathogens, adaptability to abiotic stressors. For zones of extreme viticulture in Russia, winter-hardy varieties, suitable for cultivation without covering for the winter, are needed. 

Solving these problems requires high professional qualities of breeders. Currently, the total number of researchers working in seven scientific and two educational institutions in the field of grape breeding is currently 54 people: with the degree of Doctor of Science – 7, Candidate of Science – 21, young scientists – 15.

In general, the dynamics of the number of breeders is positive. Over the past 10 years, the number of breeders has increased by 15 %. There is a positive trend in the level of qualification of breeders. The number of doctors has increased 2.5 times over the past 10 years, and the number of candidates of science has remained unchanged. The influx of young scientists increased by 1.2 times. By 2025, it is necessary to ensure continuity by increasing the number of breeders by more than 20 % in relation to 2020.

It should be noted that the training of bachelors and masters in the field of “Horticulture”, the profile “Horticulture, viticulture” is conducted by 7 agricultural universities of the Russian Federation, of which only the Kuban State Agrarian University has a group in the profile “Viticulture and wine-making” in the number of 25 full-time students and 20 part-time students.

Currently, there are no dissertation studies on grape breeding in the educational institutions that carry out postgraduate training. Only in the NCFSCHVW two postgraduate students prepare dissertations related to the grapes breeding. 

Over the past ten years (2010–2020), in eight dissertation councils working on the basis of five scientific and three educational institutions of the Russian Federation, which accept dissertations in the specialty 06.01.05 “Breeding and seed production”, have been defended six thesis works on viticulture, of which three have focused on grape breeding, including one for the degree of Doctor of Science. 

## Conclusion

Analyzing the organization and implementation of multifactor processes related to the grapes breeding and ensuring the development of the industry with varieties of domestic breeding, it is necessary to focus on:

- low share of varieties and clones of domestic breeding in the produced planting material and laying of grape plantations;
- the absence of a systemically implemented varietal and technological policy to increase the share of the most adapted to the cultivation conditions of domestic grapes varieties in industrial plantings, allowing to produce premium wines that would be of better quality compared to wines from varieties of European breeding;
- imperfection of the legal system of the protection of intellectual property, in particular, domestic breeding achievements;
- low availability of instrumental and analytical equipment for the implementation of breeding processes by modern methods that allow to speed up the creation of varieties and the selection of clones;
- insufficient number of bachelors and masters training in the direction “Viticulture” with the specialization “Breeding”;
- insufficient effective activity of post-graduate schools for targeted training of scientists-specialists and the minimum number of dissertation research defense in breeding against the background of the growing need for highly qualified personnel of scientists.

## Conflict of interest

The authors declare no conflict of interest.
